# *Borrelia burgdorferi* surface protein Lmp1 facilitates pathogen dissemination through ticks as studied by an artificial membrane feeding system

**DOI:** 10.1038/s41598-018-20208-4

**Published:** 2018-01-30

**Authors:** Juraj Koci, Quentin Bernard, Xiuli Yang, Utpal Pal

**Affiliations:** Department of Veterinary Medicine and Virginia-Maryland Regional College of Veterinary Medicine, University of Maryland, College Park, MD 20742 USA

## Abstract

In its natural infection cycle, the pathogen of Lyme borreliosis transits between a tick vector and a mammalian host. As relatively a minor fraction of spirochetes transits between the host and the vector precluding their reliable detection at early infection, artificial membrane feeders emerged as useful tools to study roles of spirochete proteins in pathogen entry, persistence, and exit through ticks. Here we report the development of a modified membrane feeder to study the role of a *Borrelia burgdorferi* surface protein called Lmp1 in spirochete transitions between the murine host and ticks. We show that our membrane feeder supports the blood meal engorgement process where ticks can acquire spirochetes from the feeder containing extremely low levels of pathogens (10^2^ cells/ml of blood). Our data revealed that in comparison to wild-type spirochetes, *lmp1* deletion mutants are significantly impaired for acquisition in naïve ticks as well as transmission from infected ticks. Taking together, our data suggest that Lmp1 plays an essential role in spirochete transitions between hosts and the vector. These studies also underscore the usefulness of artificial membrane feeding system as a valuable tool to study the role of *B. burgdorferi* gene-products in pathogen persistence in and passage through vector ticks.

## Introduction

*Borrelia burgdorferi* sensu lato is a group of diverse spirochetes causing Lyme borreliosis, which is prevalent in many parts of North America, Europe and Asia^1,2^. The pathogen circulates in an intricate enzootic cycle involving hard ticks of genus *Ixodes* and reservoir hosts, such as rodents, birds and other vertebrate hosts^3,4^. Following transmission to a vertebrate host from the tick vector, *B. burgdorferi* faces dermal innate immune insults at the bite site, represented by a variety of resident and myeloid cells^5^, antimicrobial peptides and complement^6,7^. The pathogen’s persistence in the arthropod vector, their transmission to vertebrates, as well as early survival in host environment is mediated by differential expression of several borrelial genes, including ones that encode outer surface proteins (Osp). Upon transmission into the dermis, *B. burgdorferi* interacts with the host via selected adhesins, such as decorin-binding proteins (DbpA and DbpB), fibronectin-binding protein (BBK32) and BB0347 amongst others facilitating tissue colonization and establishment of infection^8–19^. In addition, several Osp are known to facilitate *B. burgdorferi* infection in the ticks or in the host as well their transitions between tick-mammals, such as OspA, OspB, OspC, BBA64, p66, BBA57, BBA52, OspE, OspF, Elp and BB0405^20–36^. Expression of many of these *B. burgdorferi* genes encoding specific virulence determinants is regulated in a tight temporal and spatial manner using two-component systems^37–39^, in some cases facilitating microbial transmission from ticks or persistence in the host during early infection^3,40^.

As only a minuscule fraction of spirochetes are deposited in the skin, it is often difficult to ascertain whether reduced fitness of a mutant is attributed solely by a given mutation or contributed by either vector or host microbicidal immune responses in a skin, or combination of many factors. In an animal model, as it is not feasible to reliably quantify a dose-dependent spirochete transition and hence relative importance of *B. burgdorferi* gene-products in dissemination through ticks, artificial tick feeding systems are useful tools in studying transmission of tick-borne pathogens^41,42^, including additional purposes, such as studies on acaricide resistance^43^. Our previous studies showed that several *B. burgdorferi* virulence determinants are induced in infected tick and murine tissues^44,45^, one of which is Lmp1, annotated as a surface-located membrane protein of *B. burgdorferi* that plays critical roles in facilitating persistent infection in host. Lmp1 is an integral membrane protein with surface-exposed and distinct regions, including the N-terminal, middle and C-terminal domains, which play distinctive roles in spirochete persistence in a host^46^. Further analyses showed that middle region of Lmp1 binds to a host glycosaminoglycan molecule, the chondroitin-6-sulfate, facilitating attachment to mammalian cells^47^. Lmp1 transcripts are differentially expressed in murine hosts especially during early stages of infection and deletion of *lmp1* severely impairs the pathogen’s ability to persist in diverse tissues, most prominently in the heart^45,46^. Although *lmp1* is highly expressed in ticks^45^, its role in supporting vector-specific phases of spirochete life cycle remains enigmatic. Using a modified membrane feeder, in this study, we evaluated the importance of Lmp1 for spirochete entry and exit through ticks.

## Materials and Methods

### *B. burgdorferi* isolates, mice and ticks

A low-passage and infectious isolate of *Borrelia burgdorferi* B31, clone A3 and isogenic *lmp1* gene deletion mutants and *lmp1* genetically complemented isolates were used in the study^45–47^. The isolates were cultured at 34 °C in Barbour-Stoenner-Kelly medium (BSK-H) containing 6% heat-inactivated rabbit serum and checked for retention of endogenous plasmids by PCR before using in the study. For the *B. burgdorferi lmp1* gene expression experiments in feeding ticks, five-week-old female C3H/HeN mice were purchased from Charles River. Mice were subcutaneously infected with 10^5^ wild type *B. burgdorferi* (WT) mid-log growth phase *in vitro* culture and challenged with naïve nymphal ticks at 14^th^ day post infection (10 ticks/mouse, 2-3 mice/group). All animal experiments were performed in accordance with the guidelines of the Institutional Animal Care and Use Committee and Institutional Biosafety Committee of the University of Maryland, College Park, who approved all experimental protocols used in the current manuscript. Feeding ticks (48 h, 60 h and fully replete) were collected and used for the qRT-PCR analysis. One-month old nymphal ticks from a single batch used in this study were maintained in the laboratory as detailed^45^.

### Development of an *in vitro* membrane feeder

A membrane feeder was adapted using silicone membrane as described^42,43^ with following modification. The membrane feeder consisting of Ecoflex super soft silicone rubber 00–10 (Smooth-On, Inc.) was prepared using manufacturer’s instruction with the addition of hexane (2 ml into 10 ml silicone) and upon thorough stirring, the mixture was spread over the 40 µm thick lens cleaning paper (Tiffen) with a squeegee and thinned nearly to the embedded paper in order to obtain a thickness of about 50 µm. To create a feeding capsule, cured silicone membrane was glued to an acrylic plastic tube (1.25″ diameter, 2″ height) (ePlastics) with Elastosil silicone glue E4 (Wacker Chemie AG). After overnight curing, the feeding capsule was checked for a leakage by immersing into 70% ethanol for at least 15 min, dried and treated with a deer hair scent extract (~10 mg low volatility mixture (LVM)/ml) as previously described^43^. Briefly, about 25 g of deer hair cut to small pieces was soaked in 125 ml of dichloromethane (DCM, Fisher Scientific) for 30 min and repeated two more times. All three DCM solutions were combined and spun at 1000 g for 30 min. Supernatants were removed, aliquoted into 1.5 ml centrifuge tubes and concentrated in Eppendorf Vacufuge concentrator to about a third of their original volume. Concentrated stock extract was pooled, aliquoted to 1 ml and stored at −80 °C. Stock extract was adjusted to 100 mg LVM/ml by evaporation of 1 ml on a weighing scale and subsequently diluted to 10 mg LVM/ml working solution with DCM. Hundred microliters of the working solution was applied in each capsule and evaporated at room temperature overnight. After the scent treatment, the capsule equipped with an external acrylic ring was filled with ticks and inserted into a 6-well plate well containing 3 ml of a fresh defibrinated bovine blood purchased from Lampire Biological Laboratories, further supplemented with glucose (10 mM) and ATP (1 mM). Blood was changed every 12 to 14 hours. The tick feeding assembly was placed inside of a water bath incubator at 37 °C with 100% humidity with 16:8 light:dark period.

### Quantitative RT-PCR

Measurements of *B. burgdorferi* levels in ticks or in bovine blood meal were performed using quantitative RT-PCR (qRT-PCR) as described earlier^45^. Briefly, total RNA was extracted from ticks or blood samples using TRIzol (ThermoFisher Scientific), treated with RNase-free DNaseI (NEB), and then reverse transcribed to cDNA using the Superscript VILO cDNA synthesis kit (ThermoFisher Scientific). The relative spirochete levels were assessed by qRT-PCR measuring the *flaB* transcript level using primers, with a detection limit of approximately 10 spirochete cells (F: 5′TTG CTG ATC AAG CTC AAT ATA ACC A3′; R: 5′TTG AGA CCC TGA AAG TGA TGC3′) and normalized against tick *β-actin* using primers (F: 5′AGA GGG AAA TCG TGC GTG AC3′; R: 5′CAA TAG TGA TGA CCT GGC CGT3′) as described^45^. The relative levels of *B. burgdorferi lmp1* in ticks during feeding were measured by qRT-PCR using primers (F: 5′GAA ATT GCC AAC AGT AGT CC3′; R: 5′GGT CTT CTT CTT TTG GGT TT3′) and normalized against *B. burgdorferi flaB* transcripts using primers as previously described^45^. To test the efficiency and exclude nonspecific amplification of the primer pairs, the qRT-PCR amplification in each well was followed by melt-curve analysis. The individual ticks were processed separately.

### *In vitro* tick feeding studies to assess transmission from infected ticks or acquisition in naïve ticks

As *lmp1* deletion mutants (∆Lmp1) display impaired infectivity in mice^45,46^, and thus cannot be acquired in ticks, we generated spirochete-infected ticks using microinjection, having equal levels of wild type strain (WT) or ∆Lmp1 as previously described^48^. In the initial experiments, we used *B. burgdorferi*-infected ticks or naïve (control) ticks. After the tick placement on the membrane feeder, aliquots (250 µl) of the blood meal collected at 4^th^, 6^th^ and 8^th^ day of tick feeding, which were directly processed for qRT-PCR or cultured in BSK medium for 14 days and screened for viable spirochetes via dark-field microscopy. Fifty microinjected ticks were placed into each of the membrane capsule feeders. At day 4, 6 and 8^th^ of tick feeding, a 250 µL aliquot of bovine blood sample was collected and stored at −80 °C. The efficiency of tick transmission was assessed by measuring *B. burgdorferi* burden in blood and ticks using qRT-PCR analysis. For the *in vitro* acquisition experiments, prior to addition of WT, ∆Lmp1 or *lmp1* genetically complemented isolates (*lmp1* Com) into bovine blood, naïve nymphal ticks (50 ticks/capsule) were allowed to attach and initiate blood meal engorgement, until the feces were visible suggesting an active feeding process. Then the cultured-grown WT, ∆Lmp1 or *lmp1* Com *B. burgdorferi*, collected at mid-log phase were added to a freshly changed bovine blood sample as a ratio of 10^2^, 10^3^ and 10^4^ spirochetes/ml (WT or ∆Lmp1) or 10^3^ spirochetes/ml (*lmp1* Com) of blood in a total volume of three ml. Ticks were allowed to feed to full repletion, which may last until 8 days, collected, weighted and stored at −80 °C. The spirochete burden acquired by the feeding ticks was measured by qRT-PCR^45^.

### Statistical analysis

The data were represented as median with error bars indicating 95% confidence interval (CI) or as mean with error bars indicating standard mean of error (SEM). Statistical differences were measured by using the non-parametric Mann Whitney two-tailed test in Prism 7 (GraphPad Software, Inc.).

## Results and Discussion

**Figure 1:**
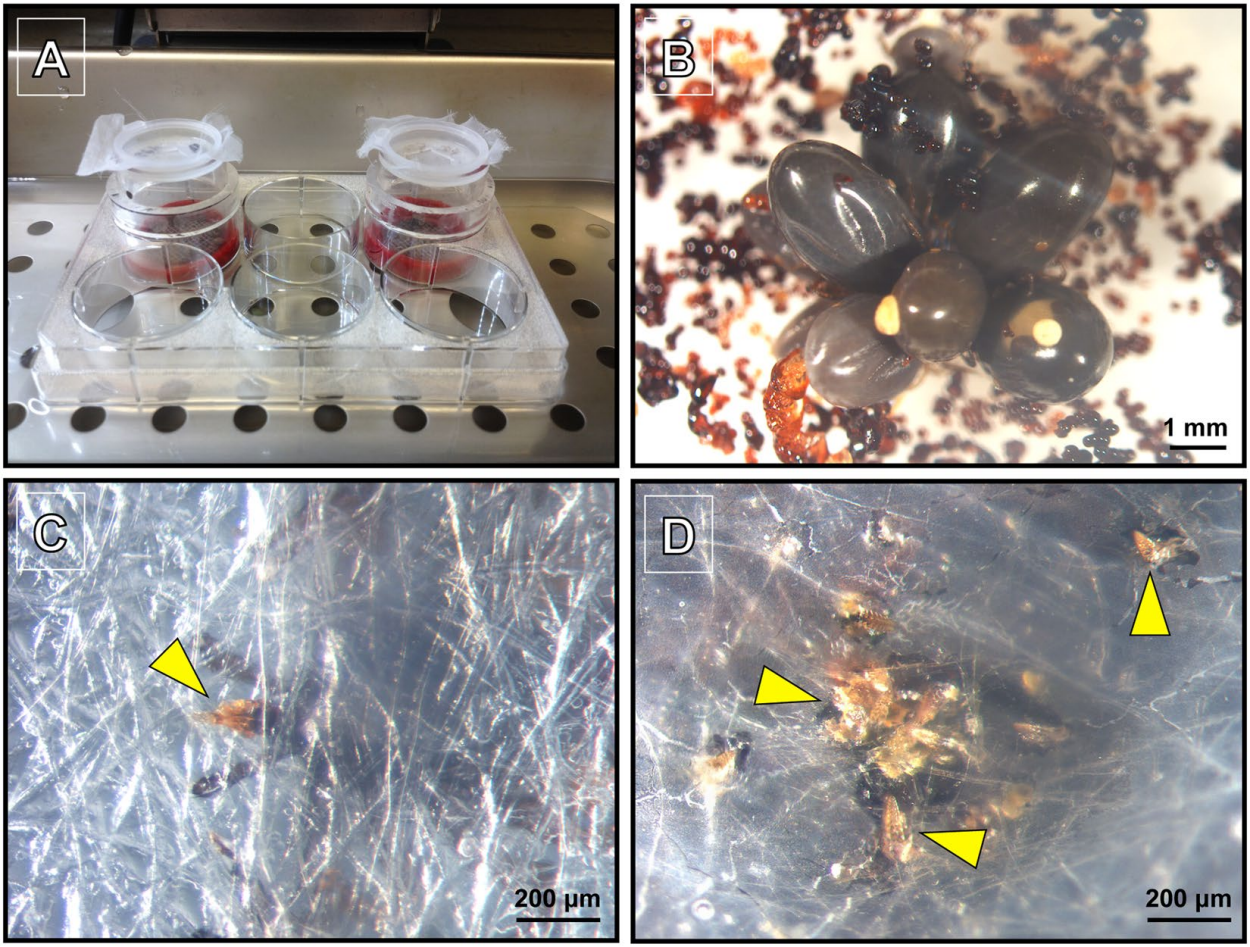
Artificial membrane feeding system. (**A**) System setup in the water bath, consisting of capsule containing ticks attached on a silicone membrane treated with a deer hair extract, dipped in the blood in a 6-well plate. (**B**) Close-up image of nymphal tick feeding cluster inside the feeding capsule. Abundant feces are apparent underneath the ticks. (**C**) Outside view of the nymphal tick hypostome protruding from the silicone membrane, indicated by a yellow arrowhead. (**D**) Outside view of the nymphal tick hypostome cluster protruding from the silicone membrane, indicated by the yellow arrowheads.

We modified the existing artificial tick feeding systems using a combination of previous approaches^42,43^, primarily via implementation of silicone membrane of reduced thickness and flexibility as well as treatment with volatile compounds extracted from deer hair for potentially better attachment and feeding efficiency. Previous studies showed that utilization of a bovine hair extract as olfactory stimulus improved tick feeding efficiency *in vitro*^43^. However, in our membrane feeder system, we observed that substitution of bovine hair extract with deer hair achieved consistently higher and faster tick attachment rate. This modification was successfully used with both adult and nymphal ticks (data not shown). The setup of the artificial tick feeding system is presented in Fig. 1. A representative example of successfully fed ticks were shown, where several hypostomes through the thin silicone membrane are clearly visible towards the underside of the silicon membrane (Fig. 1D) illustrating the flexibility and thickness of the membrane used in artificial feeding system. Given the average length of hypostome is 170 µm in *Ixodes* nymphs^43^, our membranes are considerably thinner, and potentially, could also be used also for feeding larval *I. scapularis* ticks, as previously demonstrated by other studies^42^.

**Figure 2:**
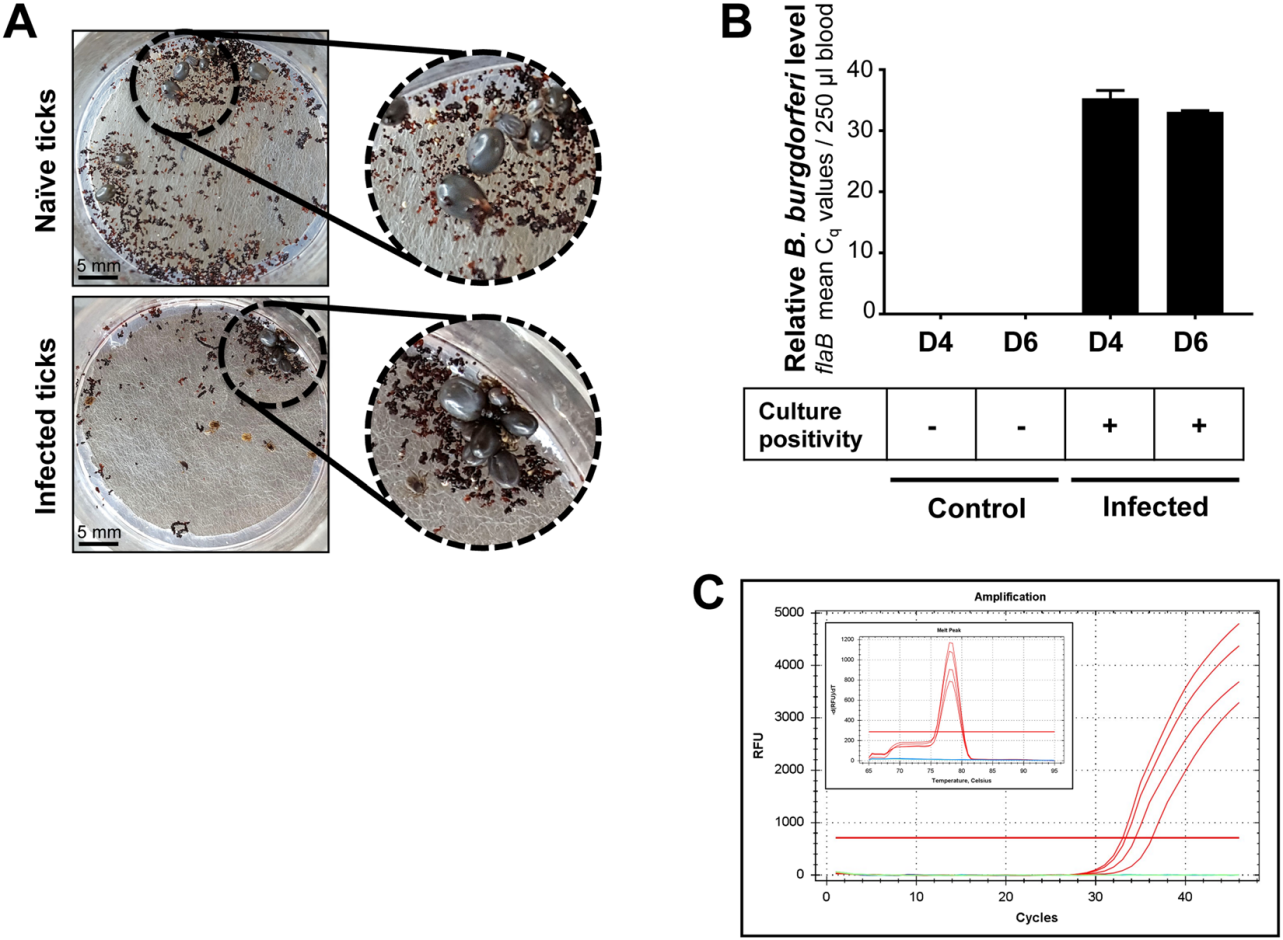
Transmission of *B. burgdorferi* in the blood using artificial membrane feeding. (**A**) Feeding of naïve (control) and wild type infectious *B. burgdorferi*-infected nymphal ticks on the membrane feeder. (**B**) Relative pathogen burden (as expressed by mean Cq values) obtained with qRT-PCR using primers targeting *flaB*. Error bars are showing ±SEM. The blood meal samples were collected between days 4–6 after feeding of naïve or infected ticks and used for detection of spirochetes via qRT-PCR or cultured to detect spirochetes via dark-field microscopy. (**C**) Amplification and specificity of spirochete detection via qRT-PCR. Amplification curves in blood samples are shown as follows: *B. burgdorferi*-infected ticks (red), naive control ticks (blue) and no DNA (template) controls (green). Inset graph shows a typical melting peak specific for *flaB* amplification.

**Figure 3:**
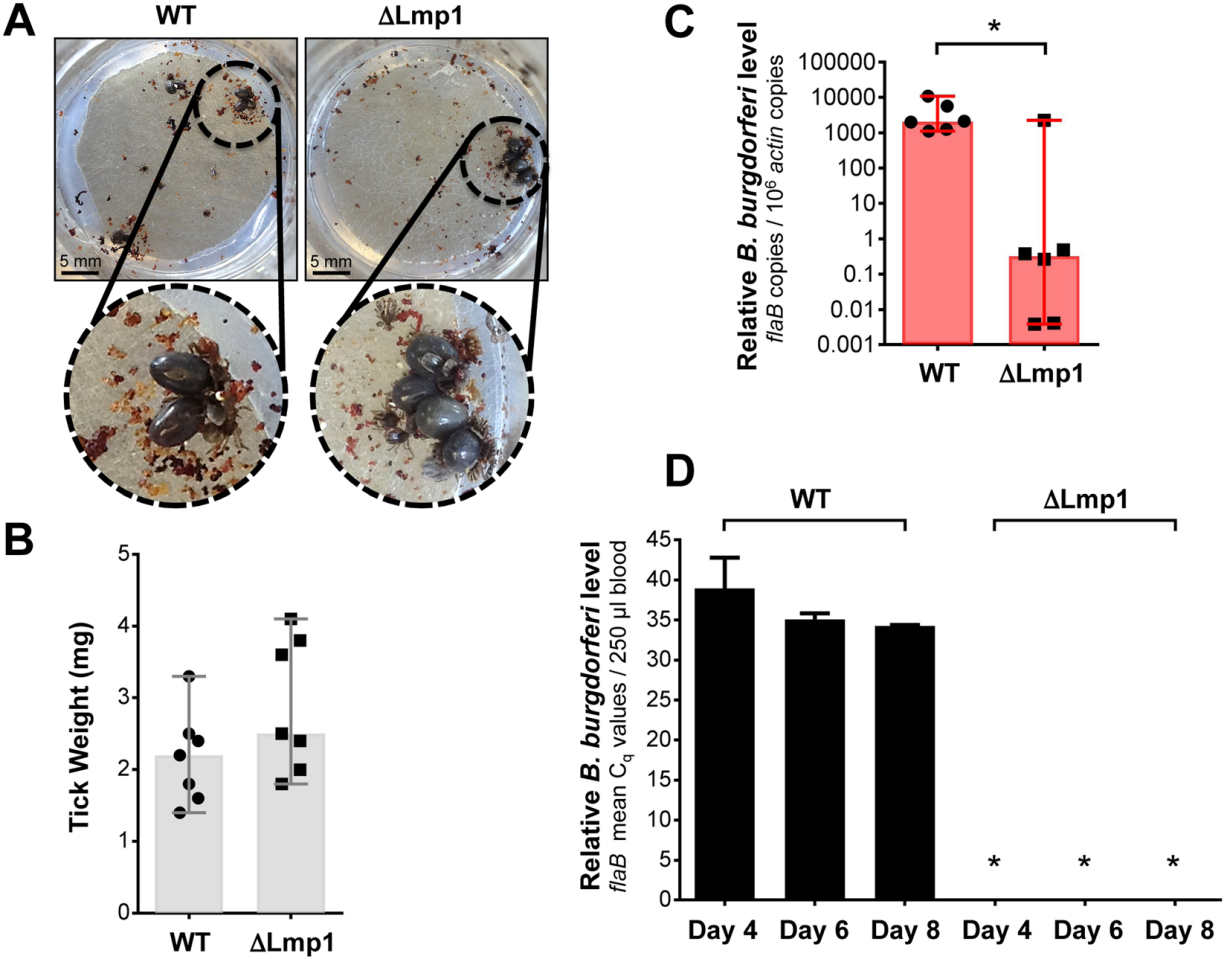
Transmission of wild type (WT) and *lmp1* deletion mutant (∆Lmp1) *B. burgdorferi* using artificial membrane feeding. (**A**) Representative images show WT-infected and ∆Lmp1-infected nymphal ticks feeding on silicone membrane. (**B**) Nymphal tick weight after transmission. The weight of the replete ticks was measured. There was no statistically significant difference in weights between WT and ∆Lmp1-infected ticks (p > 0.05). Each dot represents a fully engorged tick with bars showing median weight (n = 7). Error bars are showing 95% CI. (**C**) *B. burgdorferi* burden in fully engorged nymphal ticks. After the WT-infected and ∆Lmp1-infected ticks completed feeding on blood meal, the spirochete burden was directly detected by qRT-PCR. Significant median decrease of ∆Lmp1 burden in ticks was detected compared to WT (p < 0.05) (n = 6). Error bars are showing 95% CI. (**D**) Spirochete burden in blood after nymphal tick transmission. The *B. burgdorferi* burden was detected in 250ul of blood sample after either WT-infected or ∆Lmp1-infected ticks fed on blood meal in artificial feeding chamber using qRT-PCR (n = 2). *Undetectable or extremely low level of ∆Lmp1 was transmitted to blood compared to WT isolate. Spirochete burden is expressed as mean ± SEM. Tick data shown in this figure originated from nymphal ticks collected after detachment or after day 8 at the completion of an experiment.

We next sought to use our artificial tick feeding system to study the role of a *B. burgdorferi* antigen in pathogen egress from infected ticks. Initial experiments on the assessment of spirochete transmission involved naïve (control ticks) and *B. burgdorferi*-infected ticks in order to assess minimum infestation limit (number of ticks needed for reliable detection of spirochete transmission into a blood meal in our feeder system) and subsequent detection using qRT-PCR and culture. This is necessary as, particularly during natural transmission, only a limited number of pathogens migrate to the salivary glands^49–51^ and are ultimately deposited in the host dermis. We observed that engorgement of as few as six infected ticks were sufficient for measurement of reliable transmission of spirochetes that could be detected in bovine blood samples (Fig. 2A,C). Similarly, direct evidence of transmitted spirochetes was also obtained by culture of blood meal samples in BSK media showing viable spirochetes are transmitted from spirochete-infected ticks. On the contrary, control blood meal used for the engorgement of naïve ticks remained culture negative, as expected (Fig. 2B,C). Previous study has demonstrated that one of the surface proteins of *B. burgdorferi*, annotated as surface-located membrane protein 1 or Lmp1, is a microbial virulence determinant associated with the pathogenesis of Lyme disease^45–47^. Deletion of Lmp1, or a specific region of the protein severely impairs the pathogen infectivity in murine host^45–47^. However, no information is available for the biological significance of Lmp1 supporting spirochete persistence in ticks or their transmission through ticks. We therefore tested the effects of *lmp1* deletion on tick transmission using our artificial membrane feeder. The ticks microinjected with equal levels of either wild type (WT) or *lmp1* mutants (∆Lmp1) cells were allowed to feed on blood meal in the feeding chamber. The results showed that the attachment and frequency of ticks feeding to repletion was comparable between WT and ∆Lmp1-infected ticks (Fig. 3A). The weight of fully replete ticks was also similar (Fig. 3B). We assessed the *B. burgdorferi* level in fully replete ticks by qRT-PCR. Although we infected the ticks with the same number of spirochetes, the ∆Lmp1 *B. burgdorferi* burden in ticks was significantly lower than that of WT (Fig. 3C), indicating the persistence of ∆Lmp1 spirochetes is impaired in ticks. Despite we detected a low level of ∆Lmp1 cells in ticks, there was no transmission in blood meal feeder, at least with the current parameters used in our study, suggesting that Lmp1 function is required for spirochete egress from ticks (Fig. 3D).

**Figure 4:**
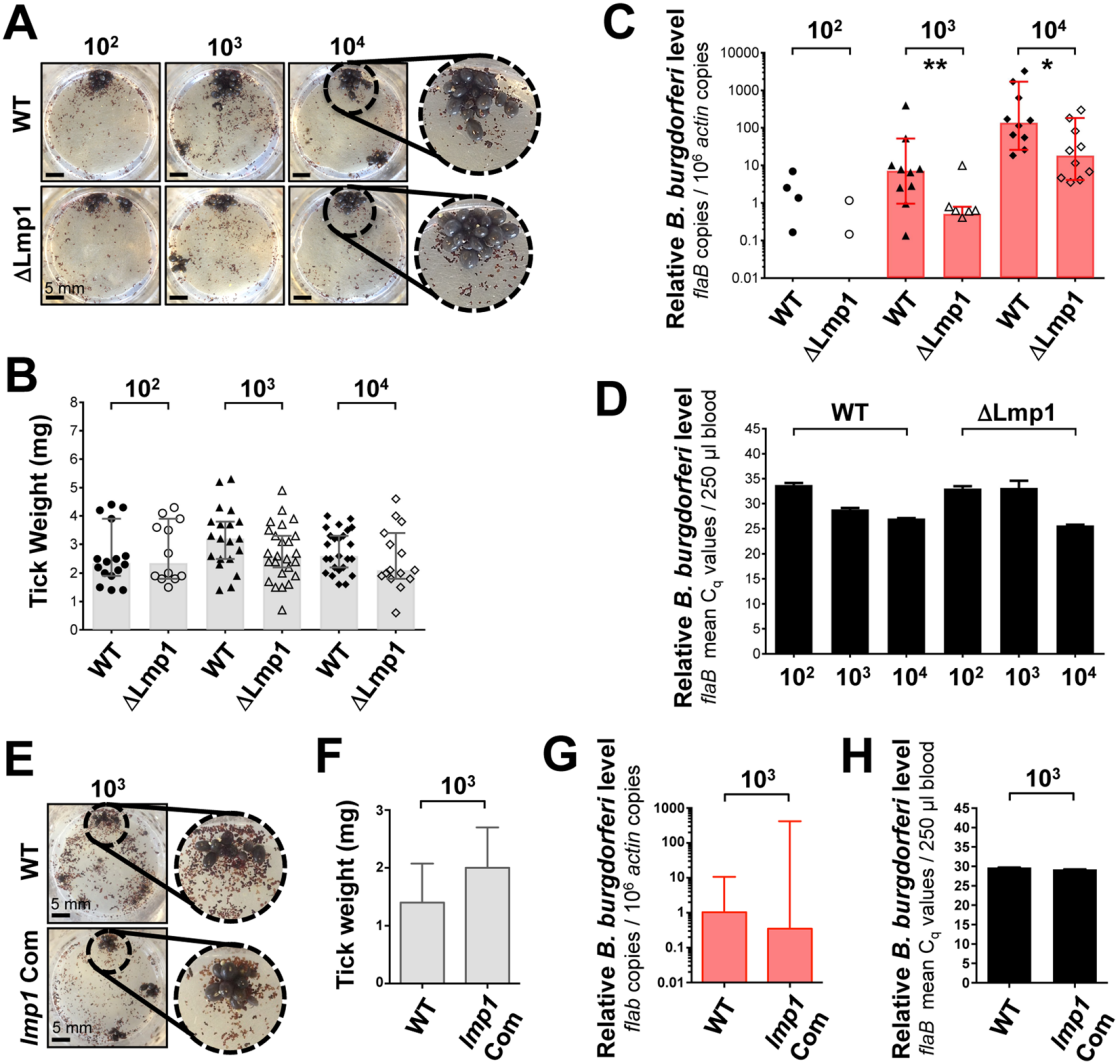
Acquisition of wild type (WT), *lmp1* deletion mutant (∆Lmp1) and *lmp1* complemented strain (*lmp1* Com) *B. burgdorferi* using artificial membrane feeding. (**A**) Representative images show feeding of naïve nymphal ticks on the bovine blood (3 ml), spiked with WT or ∆Lmp1 spirochetes at the concentrations of 10^2^, 10^3^ and 10^4^ cells/ml during every blood change. Scale bar, 5 mm. (**B**) Weight of engorged nymphs following acquisition of spirochetes. Grey bars indicate median weight of fully engorged ticks feeding on blood infected with different numbers of WT and ∆Lmp1 spirochetes. There was no significant difference in tick weight feeding on WT (solid dot) or ∆Lmp1 (open dot) spirochetes (p > 0.05). Each dot represents a fully engorged tick (n = 12–25). Error bars are showing 95% CI. (**C**) *B. burgdorferi* burden in nymphal ticks after acquisition. Significantly decreased median spirochete burden was directly detected by qRT-PCR after feeding on ∆Lmp1-spiked blood meal compared to WT (p < 0.05). Each dot represents a fully engorged tick (n = 10). Error bars are showing 95% CI. (**D**) Spirochete level in bovine blood meal. The WT and ∆Lmp1 level in blood meal, collected following a blood change, was detected using qRT-PCR (n = 2). There was no difference in spirochete level in blood meal during tick artificial feeding (p > 0.05). Bars represent the mean ± SEM. (**E**) Representative images showing feeding of naïve ticks on the blood (3 ml) spiked with 10^3^ of WT or *lmp1* Com spirochetes per ml during every blood change. (**F**) Weight of engorged nymphs. Median weight of fully engorged ticks (n = 20) feeding on blood infected with WT and *lmp1* Com spirochetes. Error bars are showing 95% CI. (**G**) Spirochete levels in engorged ticks. Median burden of WT and *lmp1* Com acquired in fully engorged ticks are shown. Error bars reflect 95% CI. (**H**) Spirochete level in bovine blood meal. The WT and *lmp1* Com level in blood meal, collected following a blood change, was detected using qRT-PCR (n = 2). There was no difference in spirochete level in blood meal during tick artificial feeding (p > 0.05). Bars represent the mean ± SEM. Tick data shown in this figure originated from nymphal ticks collected after detachment or after day 8 at the completion of an experiment.

The life cycle of *B. burgdorferi* involves acquisition of spirochetes from an infected host by larval ticks, which then molt to nymphal ticks and then transmit *B. burgdorferi* into a next mammalian host^3^. In our laboratory, the animal model of Lyme borreliosis^52^ has been routinely used as a common tool to study the ability of genetically modified spirochetes to be acquired by ticks and persist, and then transmit from ticks^53^. Notably, acquisition of spirochetes by a naïve tick from the infected host cannot be examined in a host where deletion of an important gene like *lmp1* in deletion mutants (∆Lmp1) display severe defects in persistence^45–47^. As the role of Lmp1 for spirochete acquisition in ticks also remained unknown, we used our membrane feeder system to study role of Lmp1 in spirochete acquisition in the vector. We allowed naïve ticks to engorge on artificial feeding chamber spiked with same number of wild type (WT) and ∆Lmp1 spirochetes in the bovine blood. Naïve ticks were allowed to feed on the membrane feeder containing bovine blood with varied concentrations of spirochetes ranging from 10^2^ to 10^4^ cells/ml. As shown in Fig. 4A, a comparable tick attachment or frequency of ticks feeding to repletion was observed between all groups, indicating that nymphal engorgement on a blood meal containing wild type or mutant spirochetes do not affect tick feeding process. This is further confirmed by the comparable weight of fully replete ticks. The median weight of ticks was variable, in range of 2.1 to 3.2 mg, but did not vary significantly among the groups of WT and ∆Lmp1 spirochetes (Fig. 4B). Analysis of spirochete burden in fed ticks (Fig. 4C) revealed the following notable results: first, even with the lowest concentration of spirochetes (10^2^ cells/ml) added in the membrane feeder, *B. burgdorferi* were detectable in the engorged ticks for both WT (40%) and ∆Lmp1 (20%). Second, the acquisition of ∆Lmp1 spirochetes was severely impaired, even reflected in the lowest spirochete concentration (10^2^/ml) (Fig. 4C). The deficiency of ∆Lmp1 cells in acquisition by ticks was more pronounced in all higher concentrations (10^3^ or 10^4^ cells/ml) (Fig. 4C). Third, a similar level of WT and ∆Lmp1 spirochetes persisted in the feeding chamber suggesting a persistence defect of mutants only in the ticks but not in the feeder which does not appear to affect the survival of the *B. burgdorferi* (Fig. 4D). As shown in our previous studies^45–47^, the observed phenotypic defects of *lmp1* mutants could be restored by the genetic complementation of the gene. Use of *lmp1*-complemented (*lmp1* Com) or WT spirochete-infected ticks in the membrane feeder system reflected comparable tick attachment efficiency (Fig. 4E), tick engorgement rates (Fig. 4F), as well as similar levels of *lmp1* Com and WT spirochete acquisition in fed ticks (Fig. 4G,H). Therefore, these results strongly indicate that the mutant defects for impaired survival in ticks is due to the loss of *lmp1* function but not other aberrant effects of gene manipulation process. Taken together, these results further highlight the utility of the *in vitro* tick feeding system as a reliable tool to experimentally control spirochete numbers in blood meal, lacking active components of the host immunity, and study the tick acquisition based on a comparable level of *B. burgdorferi* for all groups of wild type and genetically attenuated mutant cells. These data also suggest that Lmp1 is critical for tick acquisition of *B. burgdorferi*. The gene-product therefore not only facilitates spirochete infectivity in mammalian host^45–47,54^ but also likely supports *B. burgdorferi* persistence in tick vector. This contention is further supported by the *in vivo* assessment of *lmp1* expression in ticks feeding on mice infected with wild type *B. burgdorferi* (Fig. S1). We observed that *lmp1* expression in ticks progressed with feeding with highest expression noted at 60 hours of host attachment followed by a decrease in fully replete ticks (Fig. S1). Therefore the observed persistence defect of *lmp1* mutants in feeding ticks is likely to be related to an unknown biological function of Lmp1 in feeding ticks. Taken together, these observations further underscore the role of Lmp1, as a multi-domain protein, for multiple functions in spirochete biology and infectivity, involving ones supporting spirochete persistence in the vector.

In conclusion, our data suggest that membrane feeders are useful tools to address functions of spirochetes gene-products during entry, persistence and transmission through ticks. Such feeding interface, in the absence of a live host, offers a useful environment to study roles of host and/or pathogen molecules during vector-specific phase of spirochete life cycle. The unique opportunities offered by these membrane feeders, for example, via addition of a defined molecule (such as a host factor, or antibodies or reagents of gene silencing) or mutant spirochetes lacking a gene added to tick blood meal, represent a novel approach for systematic dissection of the role of a target molecule in spirochete persistence in the feeding tick gut. In our current study, we provide a novel use of one of these membrane feeders to study the role of a spirochete protein showing an important role for *B. burgdorferi* Lmp1 supporting pathogen acquisition and transmission by ticks. Although how Lmp1 supports spirochete survival in ticks remains elusive, this multi-domain and multi-functional antigen has been implicated in several biological functions and considered as a vaccine candidate^55^ to combat Lyme borreliosis. Further utilization of these membrane feeders will allow us to dissect roles of additional *B. burgdorferi* gene-products like Lmp1, especially many intriguing surface proteins of undefined function for their biological significance in the pathogen’s life cycle.

## Electronic supplementary material

Supplementary information
