# Supplementary material for: Borrelia burgdorferi surface protein Lmp1 facilitates pathogen dissemination through ticks as studied by an artificial membrane feeding system
Source: Sci Rep. 2018 Jan 30;8:1910. doi: 10.1038/s41598-018-20208-4 (PMC5790009; doi:10.1038/s41598-018-20208-4)
Supplement: Supplementary file 1 — Supplementary information [file 41598_2018_20208_MOESM1_ESM.pdf]

Supplementary Information for the manuscript entitled

Borrelia burgdorferi surface protein Lmp1 facilitates pathogen dissemination through ticks as studied by an artificial membrane feeding system

Juraj Koci, Quentin Bernard, Xiuli Yang and Utpal Pal

The supplementary information contains:
One Supplementary Figure and Legends to Supplementary Figure

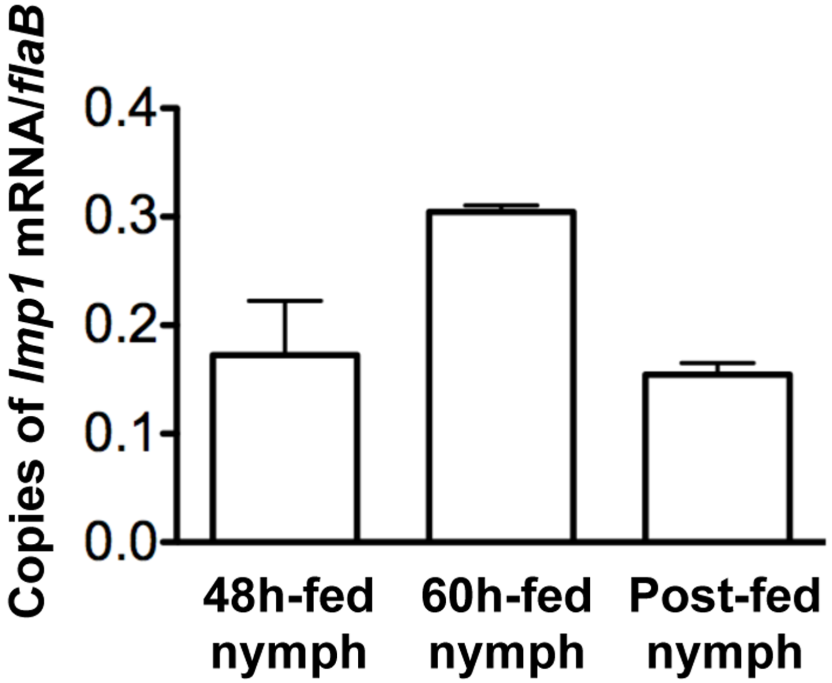

Copies of *Imp1* mRNA/*flaB*
0.4
0.3
0.2
0.1
0.0
48h-fed nymph
60h-fed nymph
Post-fed nymph

Legends to Supplementary Figures

Fig. S1. *Lmp1* expression in feeding nymphal ticks. The relative expression of *lmp1* is presented as copies of *lmp1* transcripts per 1000 copies of *flaB* transcripts, measured using qRT-PCR in individual ticks collected at multiple time points during feeding on mice 14 days after infection with wild-type *B. burgdorferi*. Bars represent the mean ± SEM of at least two experimental replicates.
